# HOXA5 Expression Is Elevated in Breast Cancer and Is Transcriptionally Regulated by Estradiol

**DOI:** 10.3389/fgene.2020.592436

**Published:** 2020-12-15

**Authors:** Imran Hussain, Paromita Deb, Avisankar Chini, Monira Obaid, Arunoday Bhan, Khairul I. Ansari, Bibhu P. Mishra, Samara A. Bobzean, S. M. Nashir Udden, Prasanna G. Alluri, Hriday K. Das, Robert Matthew Brothers, Linda I. Perrotti, Subhrangsu S. Mandal

**Affiliations:** ^1^Department of Chemistry and Biochemistry, The University of Texas at Arlington, Arlington, TX, United States; ^2^Department of Psychology, The University of Texas at Arlington, Arlington, TX, United States; ^3^Department of Radiation Oncology, The University of Texas Southwestern Medical Center, Dallas, TX, United States; ^4^Department of Pharmacology and Neuroscience, University of North Texas Health Science Center, Institute for Healthy Aging, Fort Worth, TX, United States; ^5^Department of Kinesiology, The University of Texas at Arlington, Arlington, TX, United States

**Keywords:** HOXA5, breast cancer, estradiol, gene expression, estrogen receptors, chromatin

## Abstract

HOXA5 is a homeobox-containing gene associated with the development of the lung, gastrointestinal tract, and vertebrae. Here, we investigate potential roles and the gene regulatory mechanism in HOXA5 in breast cancer cells. Our studies demonstrate that HOXA5 expression is elevated in breast cancer tissues and in estrogen receptor (ER)-positive breast cancer cells. HOXA5 expression is critical for breast cancer cell viability. Biochemical studies show that estradiol (E2) regulates HOXA5 gene expression in cultured breast cancer cells *in vitro*. HOXA5 expression is also upregulated *in vivo* in the mammary tissues of ovariectomized female rats. E2-induced HOXA5 expression is coordinated by ERs. Knockdown of either ERα or ERβ downregulated E2-induced HOXA5 expression. Additionally, ER co-regulators, including CBP/p300 (histone acetylases) and MLL-histone methylases (MLL2, MLL3), histone acetylation-, and H3K4 trimethylation levels are enriched at the HOXA5 promoter in present E2. In summary, our studies demonstrate that HOXA5 is overexpressed in breast cancer and is transcriptionally regulated via estradiol in breast cancer cells.

## Introduction

HOX genes are a highly conserved family of homeobox-containing genes that play pivotal roles in cell differentiation, development, and embryogenesis (Krumlauf, 1994; Zakany and Duboule, 2007). The collinear expression of HOX genes are critical in anterior–posterior patterning during embryogenesis (Akam, 1987; Mlodzik et al., 1988; Duboule and Dolle, 1989; McGinnis and Krumlauf, 1992; Mallo et al., 2010). In humans, there are 39 HOX genes that are classified as HOXA-D clusters (Acampora et al., 1989; Garcia-Fernandez, 2005; Holland et al., 2007; Mallo and Alonso, 2013), and they primarily function as transcription factors (Svingen and Tonissen, 2006; Plowright et al., 2009; Ladam and Sagerstrom, 2014; Zheng et al., 2015; Morgan et al., 2016). They bind to their target gene promoters via their homeodomain and control gene expression (Hanson et al., 1999; Shah and Sukumar, 2010; Boube et al., 2014; Taniguchi, 2014). Though HOX genes were originally described to exhibit collinear expression, especially during embryogenesis, recent studies demonstrate that each HOX gene may be independently regulated and expressed in various adult tissues (Taylor et al., 1998; Neville et al., 2002; Takahashi et al., 2004; Dunwell and Holland, 2016; Rux and Wellik, 2017). Additionally, HOX gene expressions are misregulated in a variety of cancers (Grier et al., 2005; Shah and Sukumar, 2010; Gray et al., 2011; Bhatlekar et al., 2014). HOX genes are associated with cancer cell proliferation, angiogenesis, and tumor growth (Hayashida et al., 2010; Ansari et al., 2011a,b; Shrestha et al., 2012; Hussain et al., 2015; Deb et al., 2016). HOX gene expression is increasingly recognized as a potential biomarker and target of therapeutic intervention. For example, HOXB9, a key player in mammary gland development, is upregulated in breast cancer, regulates growth and angiogenic factors, and is a potential biomarker (Ansari et al., 2011b; Shrestha et al., 2012). Similarly, another HOX gene, such as HOXC6, implicated in mammary gland development and breast cancer, regulates tumor growth factors and induces 3-D-colony formation (Ansari et al., 2011a; Hussain et al., 2015). HOXB7 overexpression is linked with cell proliferation, neoplastic transformation, and tumorigenesis (Wu et al., 2006; Nguyen Kovochich et al., 2013). Spinal cord associated HOXA10 expression is upregulated in breast cancer and is regulated by estradiol (Chu et al., 2004). Thus, HOX genes are emerging as major players in gene regulation and cancer; however, their detailed functions and gene regulatory mechanism remains elusive.

Homeobox-containing gene HOXA5 is a critical player in the development of lung, gastrointestinal tract, spleen, kidney, and vertebrae (Larochelle et al., 1999; Jeannotte et al., 2016). It regulates the expression of various proteins in conjunction with other paralogs during the ontogeny of normal development (Larochelle et al., 1999; Jeannotte et al., 2016). Studies also demonstrate that HOXA5 expression plays a critical role in development of the murine central nervous system (Joksimovic et al., 2005). In addition to its critical role in development, HOXA5 expression is dysregulated in breast epithelium and is linked to breast cancer biogenesis (Raman et al., 2000a,b). Here, we investigate if HOXA5 expression is associated with breast cancer and study its transcriptional regulatory mechanism in breast cancer cells. We demonstrate that HOXA5 expression is elevated in breast cancer, and its expression is regulated by estradiol (E2).

## Experimental Section

### Cell Culture, Antisense Oligonucleotide (ASO)-Transfection, and E2 Treatments

CCL228 (colon cancer), H358 (lung cancer), HeLa (cervical cancer), JAR (choriocarcinoma placenta), MCF10 (normal breast epithelial), MCF7 (ER-positive breast cancer), T47D (ER-positive breast cancer), and MDA-MB-231 (ER-negative breast cancer) cells (from ATCC) were cultured in DMEM that was supplemented with 10% fetal bovine serum (FBS), 2 mM L-glutamine, and 1% penicillin/streptomycin (100 units and 0.1 mg/mL, respectively) (Wang et al., 2010; Ansari et al., 2011a, 2012a; Shrestha et al., 2012; Kasiri et al., 2013; Bhan et al., 2014a; Hussain et al., 2015; Deb et al., 2016).

For the estrogen treatment, MCF7 cells were cultured in phenol red–free DMEM-F-12 supplemented with 10% charcoal-stripped FBS for at least 3 generations. Cell were then treated with 17β-E2 for 6 h and then subjected to RNA and protein extraction.

All the ASOs were synthesized commercially from IDT-DNA. MCF7 cells (grown in phenol red–free DMEM-F-12 supplemented with 10% charcoal-stripped FBS) were transfected with ASOs using iFECT transfection reagent (KD Medicals, Inc.) with antisense oligonucleotides for 48 h (Ansari et al., 2011a; Kasiri et al., 2013). ASO-transfected cells were then treated with 1 nM E2 for 4 h followed by RNA and protein extraction for further analysis.

### Immunohistological Analysis of Breast Cancer Tissue Microarray

Breast cancer tissue microarrays (US Biomax Inc) were immunostained with DAB staining using HOXA5 antibody as described by us previously (REF). Microarray slides was initially deparaffinized by immersing in xylene (twice, 10 min each), then sequentially in 100, 95, and 70% ethanol (5 min each) and then incubated in 0.1 M sodium citrate buffer (at 95°C, 15 min) for antigen retrieval. For the immunostaining, the slide was immerged in 3% H_2_O_2_ (15 min), washed with PBS (x2), blocked with buffer containing donkey serum, and then incubated with HOXA5 antibody (overnight). Slides were washed thrice (PBS) and then incubated (1.5 h) with donkey secondary antibody (biotinylated). Slides were then washed and incubated with avidin–biotin complexes (1.5 h), again washed with PBS, followed by washes with 0.1 M Tris-HCl (pH 7.4, twice). The slide was subjected to peroxidase labeling (incubated with a DAB substrate, Vector Laboratories), dehydrated (immersed sequentially in 70, 95, and 100% ethanol), cleaned (incubated in citrisolv agent), and mounted using DPX mounting solution. Slides were analyzed under a microscope (Nikon Eclipse TE2000-U, Japan).

### RNA and Protein Extraction, RT-PCR, and Western Blots

RNA extraction: cells were resuspended in DEPC-treated buffer A (20 mM Tris-HCl, pH 7.9; 1.5 mM MgCl_2_; 10 mM KCl and 0.5 mM DTT; 0.5 mM EDTA), incubated on ice (10 min), and then centrifuged. The supernatant was subjected to phenol-chloroform extraction. RNA was precipitated using ethanol. The precipitated RNA was dissolved in DEPC-treated water, quantified, and then reverse-transcribed into cDNA as described by us previously (500 ng RNA, 2.4 μM of oligo-dT, 100 units MMLV reverse transcriptase, 1X first strand buffer, 100 μM dNTPs each, 1 mM DTT, and 20 units of RNaseOut in 25 μL total reaction volume). The cDNA was diluted to 100 μL and then analyzed using regular PCR and qPCR (PCR primer in Table 1). For the qPCR, 5 μL cDNA was mixed with 5 μL of SsoFast EvaGreen supermix (Biorad) and PCR primers (100 μM each), and then PCR-amplified in a CFX96 qPCR system. Protein extracts: Whole cell protein extracts were made by suspending the cells in whole cell extraction buffer (50 mm Tris–HCl, pH 8.0; 150 mM NaCl; 1 mM EDTA; 0.05% NP-40; 0.2 mM PMSF; 0.5 mM DTT; and 1× protease inhibitor cocktail). Proteins were quantified and analyzed by Western blotting (alkaline phosphatase method).

**Table 1:** Primers used for cloning, RT-PCR, ChIP, and antisense experiments.

**Primers**	**Forward primers (5^**′**^-3^**′**^)**	**Reverse primers (5^**′**^-3^**′**^)**
**PCR PRIMERS**		
GAPDH	CAATGACCCCTTCATTGACC	GACAAGCTTCCCGTTCTCAG
HOXA5	ACTCATTTTGCGGTCGCTAT	TTGTAGCCGTAGCCGTACCT
HOXA5-ERE1	CGAGTCCGGCTGAACGGCGG	TAGGCACCCAAATATGGGGTA
HOXA5-ERE2	TTATTTCTCCAATTGGCTAAA	CCGGCGAGGATGCAGAGGAT
ERα	AGCACCCTGAAGTCTCTGGA	GATGTGGGAGAGGATGAGGA
ERβ	AAGAAGATTCCCGGCTTTGT	TCTACGCATTTCCCCTCATC
**ANTISENSES**		
HOXA5	GTCCCTGAATTGCTCGCTCA^**^	
ERα antisense	TCCCACCTTTCATCATTCCC^**^	
ERβ antisense	GCCACACTTCACCATTCCCA^**^	
Scramble	CGTTTGTCCCTCCAGCATCT^**^	
antisense		

** *Phosphorothioate antisense oligonucleotides*.

### Chromatin Immunoprecipitation (ChIP) Assay

ChIP analyses were performed using similar procedures described by us previously (Wang et al., 2010; Ansari et al., 2011a, 2012a; Shrestha et al., 2012; Kasiri et al., 2013; Bhan et al., 2014a; Hussain et al., 2015; Deb et al., 2016). Briefly, MCF7 (treated with different conditions) cells were fixed in 4% formaldehyde (15 min), followed by quenching with 125 mM glycine and washed with PBS (twice). They were lyzed (SDS lysis buffer), and the chromatin was sheared by sonication (~150–450 bp in length, checked by agarose gel). The sonicated chromatins (precleaned using protein-G agarose beads) were immunoprecipitated with antibodies against ERα, ERβ, H3K4-trimethyl, RNAPII, MLL1, MLL2, MLL2, MLL3, CBP, p300, H4 acetylation, and β-actin, independently. The immunoprecipitated DNA fragments were purified and analyzed by PCR using promoter-specific primers shown in Table 1.

### Flow Cytometry Analysis

MCF7 cells transfected with HOXA5 and scramble ASOs (48 h) were harvested and centrifuged (1,000 × g for 5 min at 4°C). Cells were washed with PBS (cold, twice), fixed (70% ethanol/PBS, 1 h, 4°C), and stored overnight at −20°C. Cells were stained with propidium iodide (PI) by resuspending in PI solution (0.5 μg/mL, 2 h) and then analyzed by FACS (Beckman Coulter, Fullerton, CA, USA) (31).

### Animal Studies

Adult, 90-days-old, experimentally naïve, female Sprague–Dawley rats (*n* = 12) (purchased commercially) and maintained in accordance with the National Institutes of Health Guidelines for the Care and Use of Laboratory Animals as described by us previously (Betancourt et al., 2010; Bhan et al., 2014a,b; Hussain et al., 2015). Rats were anesthetized (2%−3% isoflurane-oxygen vapor mixture), ovariectomized (OVX), and allowed to recover (for 4–5 days post-surgery) as described previously (Betancourt et al., 2010; Bhan et al., 2014a,b; Hussain et al., 2015). Vaginal lavage testing was performed daily for 8 consecutive days to confirm cessation of estrous cycling (Inagaki et al., 2012). Rats were given subcutaneous injections of E2 (5 μg/kg, dissolved in peanut oil, 5 μg/mL stock) (*n* = 4) 24 and 4 h prior to sacrifice (via rapid decapitation). Mammary gland tissue was collected, flash frozen on dry ice, and stored at −80°C. RNA and proteins were extracted from mammary tissues and analyzed by RT-PCR and Western blotting (Betancourt et al., 2010; Bhan et al., 2014a,b; Hussain et al., 2015).

### Statistical Analyses

Experiments were repeated at least thrice (*n* = 3). The RT-qPCR experiments were performed in three parallel replicates and repeated at least thrice. The data presented in the figures is expressed as averages ± standard error. Statistical significance between various groups (control vs. treated) were determined using one- or two-way analysis of variance (ANOVA) followed by multiple comparisons with Dunnett correction (GraphPad Prism). Other statistical evaluations were performed using the Student *t*-test. Data was considered statistically significant when the *p*-value was <0.05.

## Results

### HOXA5 Is Elevated in Breast Cancer

**Figure 1:**
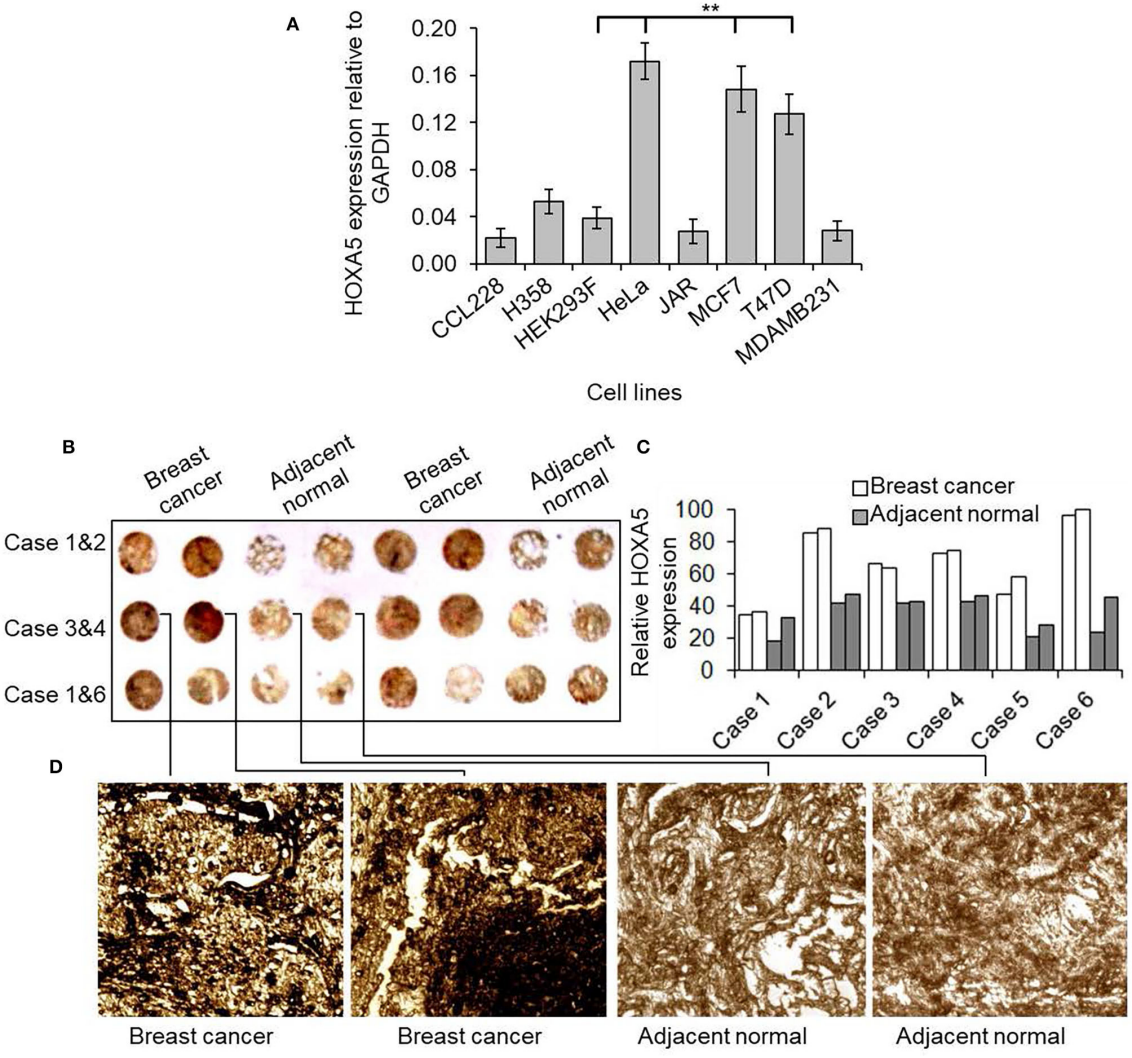
HOXA5 expression in breast cancer cells and tissues. **(A)** Comparison of HOXA5 expression in different cell lines. The total RNA was isolated from CCL228 (colon cancer), H358 (lung cancer) HeLa (cervical cancer), JAR (placental cancer), MDAMB231 (ER-negative breast cancer), T47D (ER-positive breast cancer), MCF7 (ER-positive breast cancer) cell lines and analyzed by RT-qPCR using primers specific to HOXA5 and GAPDH (loading control). HOXA5 expression relative to GAPDH is plotted. Bars indicate averages ± standard errors (*n* = 3). **denotes *p* < 0.0001 compared to HEK293F control cells **(B–D)** HOXA5 expression in breast cancer tissue. Tissue microarray slide (containing six cases of human breast cancer cases and respective adjacent normal breast tissue) was subjected to DAB staining with HOXA5 antibody. The HOXA5 expression level was quantified **(C)**. A magnified view of case 3 is in **(D)**.

In an effort to understand the potential role of HOXA5 in breast cancer, initially we examined HOXA5 expression in various cancer cells and breast cancer tissues. Briefly, RNA from different cell lines was analyzed by RT-qPCR. These analyses demonstrated that HOXA5 expression is elevated in MCF7 and T47D (ER-positive breast cancer cells) in comparison to MDA-MB-231 (ER-negative breast cancer cells) (Figure 1A). For the tissue expression analysis, a breast cancer tissue microarray (commercial) was analyzed for HOXA5 expression level. Briefly, the tissue microarray containing the breast cancer tissue and surrounding normal tissue was immunostained using a HOXA5 antibody and analyzed under a microscope. Interestingly, these analyses show that HOXA5 expression is elevated in most breast cancer tissues relative to their normal counterparts (Figures 1B–D).

**Figure 2:**
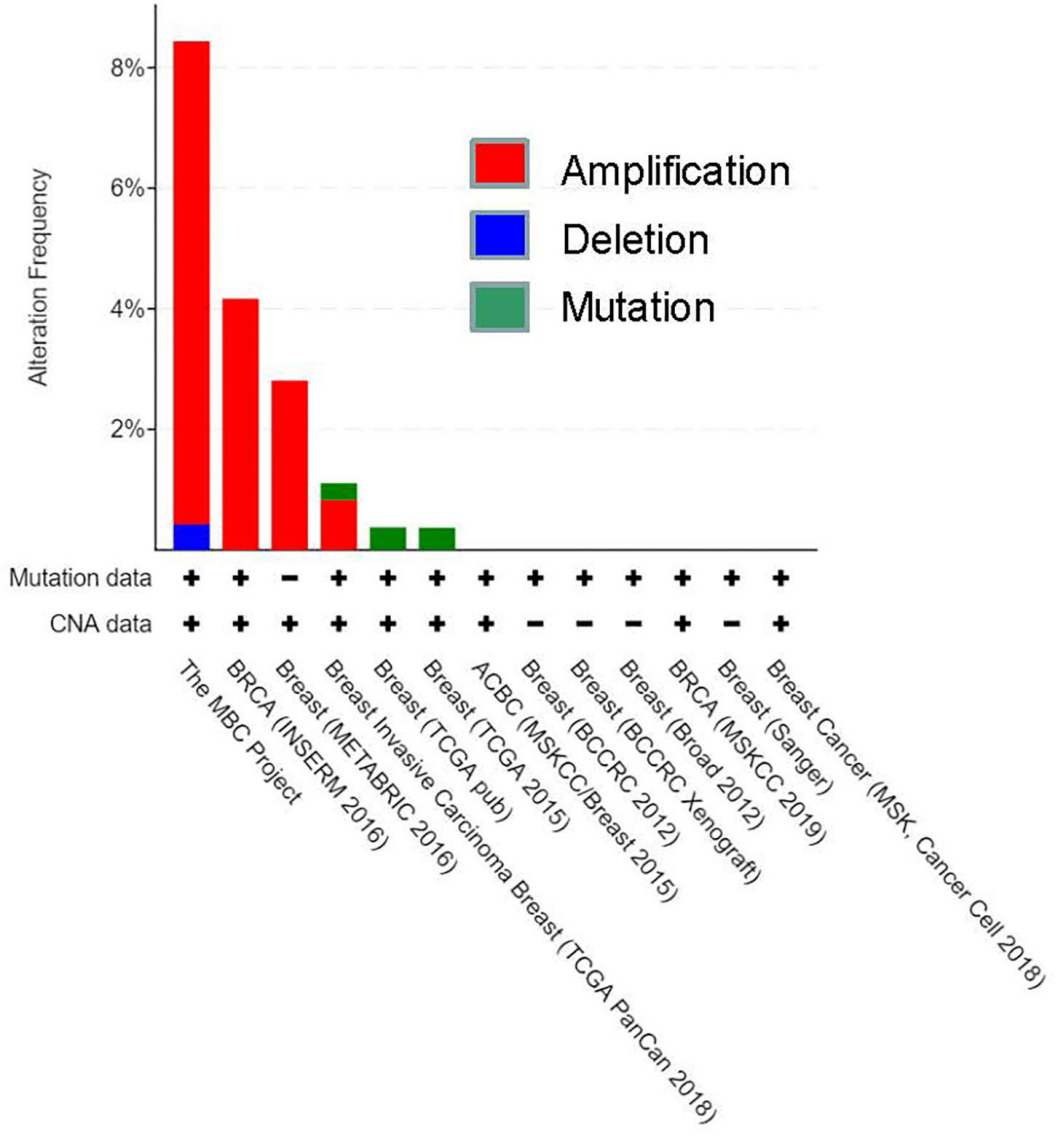
CBioportal analysis of breast cancer database for HOXA5 expression. HOXA5 expression in different types of breast cancer patients using a preexisting cancer gene expression database using a cBioPortal online data analysis tool (https://www.cbioportal.org). The data provided by cBioPortal includes mutations, deletions, and copy-number amplifications for HOXA5; CAN, copy number alteration.

To investigate further, we also examined HOXA5 expression in different types of breast cancer patients using a preexisting cancer gene expression database using cBioPortal online data analysis tool (https://www.cbioportal.org) (Figure 2). The data provided by cBioPortal includes mutations, deletions, and copy-number amplifications (Cerami et al., 2012; Gao et al., 2013). These analyses demonstrate that HOXA5 expression is significantly amplified in various breast cancer tissues, such as in MBC project, BRCA (INSERM 2016), breast (METABRIC), and invasive breast carcinoma (TCGA PanCan), further suggesting potential association of HOXA5 in breast cancer. Taken together, these observations demonstrate that HOXA5 is overexpressed at least in some subset of breast cancer and may contribute to breast cancer growth. The overexpression of HOXA5 in the ER-positive breast cancer cells suggests its potential regulation by E2.

### HOXA5 Knockdown Suppressed the Breast Cancer Cell Viability

**Figure 3:**
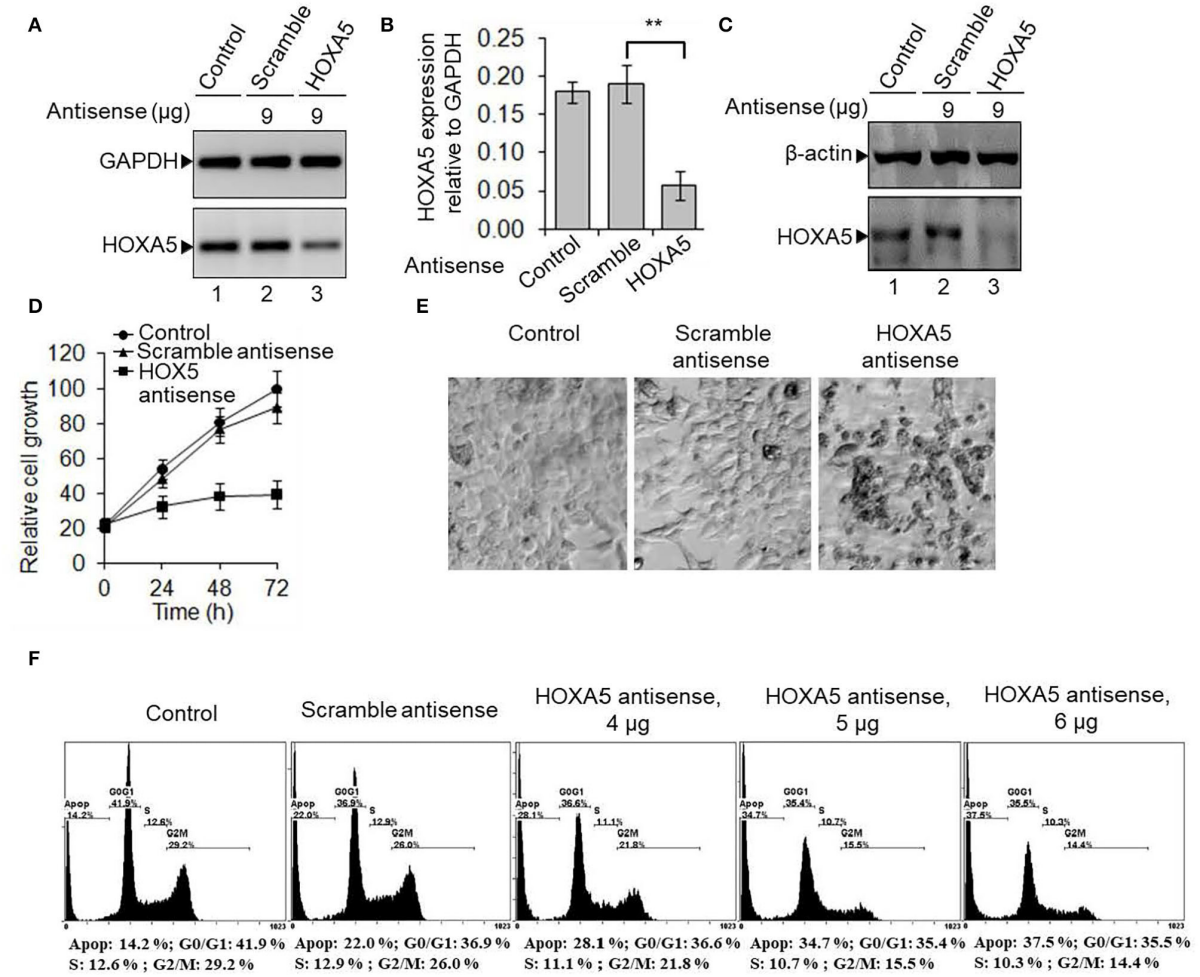
HOXA5 knockdown affects cell viability and cell-cycle progression. **(A–C)** MCF7 cells were transfected with HOXA5-specific and scramble antisense for 48 h, RNA was analyzed by RT-PCR with primers specific to HOXA5 (**A**, quantification in **B**). GAPDH was used as normalization control. Bars indicate averages ± standard errors (*n* = 3). **denotes *p* < 0.0001 compared to scramble control. Proteins were analyzed by Western blot using HOXA5 and β-actin (loading control) antibodies **(C)**. **(D,E)** Impact of HOXA5 knockdown on cell growth and viability. MCF7 cells were transfected with HOXA5-specific and scramble-antisense oligonucleotides. Live cell numbers were counted under HOXA5 knockdown conditions for varying periods of time and plotted in **(D)**. Data points indicate averages ± standard errors (*n* = 3). **denotes *p* < 0.0001 compared to scramble control. Morphologies of cells were visualized under a microscope (Nikon Eclipse TE2000-U) **(E)**. Bars indicate standard errors (*n* = 3). **(F)** Flow cytometry analysis. MCF7 cells were treated with HOXA5 and scramble antisense separately for 48 h and subjected to flow cytometry analysis. Panel 1: Control cells treated with no antisense, panel 2: cells treated with scramble-antisense, panels 3–5: cells treated with increasing concentration of HOXA5-specific antisense. The cell populations at different stages of the cell cycle are shown inside the respective panels.

As HOXA5 expression is upregulated in breast cancer, we knocked it down in MCF7 cells and analyzed its impacts on viability. HOXA5 antisense or scramble-ASO were transfected into MCF7 cells separately (48 h) followed by analysis of HOXA5 expression levels using the RNA (RT-qPCR) and protein levels (Western blots). Cells were also visualized under a fluorescence microscope or analyzed by flow cytometry. Our analyses show that HOXA5 expression is decreased upon transfection with HOXA5 ASO (Figures 3A–C), and this results in decrease in growth of MCF7 cells compared to controls (Figure 3D). Microscopic analysis shows that, upon HOXA5 knockdown, cells appear unhealthy, rounded up, and dead (Figure 3E).

To examine if HOXA5 may regulate cell-cycle progression, we knocked it down in MCF7 cells using HOXA5 ASO and analyzed it by flow cytometry. Interestingly, upon HOXA5-ASO transfection, G0/G1 and S phase cell populations were decreased with an increase in apoptotic cells compared to controls (Figure 3F). At 4 μg of HOXA5 ASO treatment, the cell population at the S (decreased from 12.9 to 11.1%) and G2/M phases (reduced from 26 to 21.8%) were reduced although about 28% of cells were apoptotic, and these affected were further pronounced at higher HOXA5 ASO concentrations (Figure 3F), suggesting the critical role of HOXA5 in regulation of cell-cycle progression.

### E@ Regulates HOXA5 Gene Expression *in vitro* and *in vivo*

**Figure 4:**
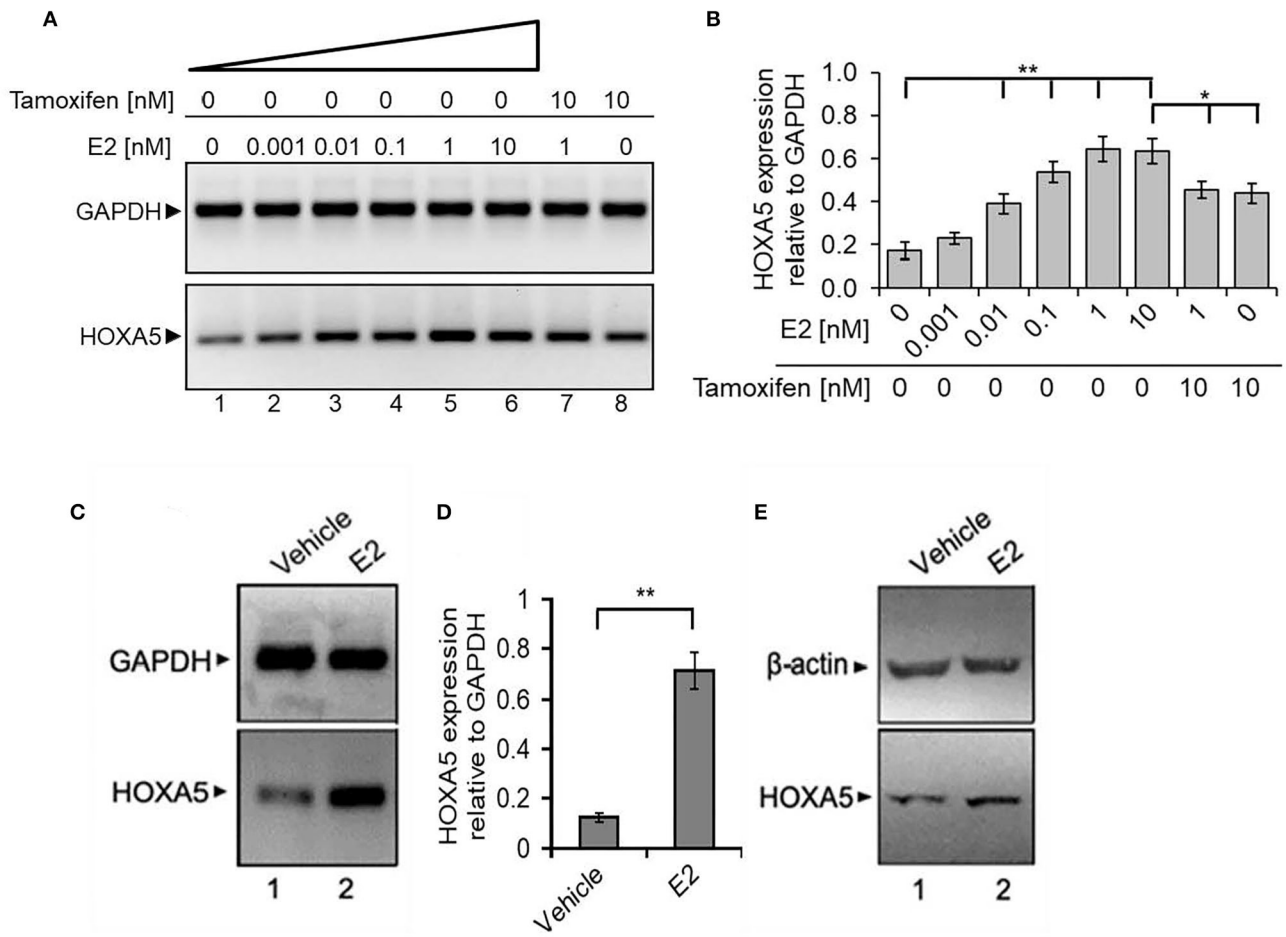
E2-induced expression of HOXA5. **(A,B)** E2-induced expression of HOXA5 in MCF7 cells. MCF7 cells (grown in phenol red–free media and charcoal-stripped FBS) were treated with varying concentrations (0–10 nM) of E2 for 6 h in the presence and absence of 10 nM tamoxifen. RNA analyzed by RT-PCR using primers specific to HOXA5 and GAPDH (control). An agarose gel analysis picture of the PCR products is shown in **(A)**, and qPCR analysis (expression of HOXA5 relative to GAPDH) is in **(B)**. Bars indicate averages ± standard errors (*n* = 3). **denotes *p* < 0.0001 compared to untreated controls; *denotes *p* < 0.001 compared to 10 nM E2. **(C–E)** Effects of E2 on HOXA5 expression in OVX rats *in vivo*. OVX female rats were treated with E2 (5 μg for 24 h) or vehicle (peanut oil/saline). RNA and protein were isolated from the mammary glands of the control and E2-treated animals. RNA was analyzed by RT-PCR using rat-specific primers for HOXA5 (quantification in **D**); GAPDH was used as a loading control. Each experiment was repeated thrice with three parallel replicates. Bars indicate averages ± standard errors, **denotes *p* < 0.0001 compared to vehicle control. Western blot analysis of the HOXA5 protein levels from the protein samples obtained from control, E2-treated mammary gland tissues of animals are shown in **(E)**. β-actin was used as the loading control. Each experiment was repeated at least thrice (*n* = 3).

As HOXA5 expression is elevated in breast cancer cells and tissues, we investigated its potential regulatory mechanism by E2 in MCF7 cells. Briefly, MCF7 cells were treated with 17β-E2. RNA was isolated and analyzed by RT-PCR for the expression of HOXA5. An antiestrogen, tamoxifen, was also used in conjunction with E2 to explore the specificity and to understand the E2-mediated regulatory mechanism of HOXA5. Interestingly, E2 treatment induced HOXA5 expression in a concentration-dependent manner (Figures 4A,B). HOXA5 expression was increased by 4.5-fold upon exposure to 1 nM of E2. Notably, treatment with the antiestrogen tamoxifen suppressed E2-induced HOXA5 expression (Figures 4A,B). These observations suggest potential regulation of HOXA5 via E2 and ERs.

To further examine the physiological significance of HOXA5 and its endocrine regulation *in vivo*, we analyzed its expression in the mammary tissue of OVX female rats treated with E2 (5 μg/kg) (Betancourt et al., 2010; Bhan et al., 2014a,b; Hussain et al., 2015). The OVX procedure was performed to minimize the effects of endogenous estrogens. Interestingly, RT-qPCR and Western blot analysis demonstrate that HOXA5 expression is elevated in the rat mammary tissues upon treatment with E2 (Figures 4C–E), indicating potential E2-mediated regulation of HOXA5 *in vivo*.

### ER Coordinates E2-Induced HOXA5 Expression

**Figure 5:**
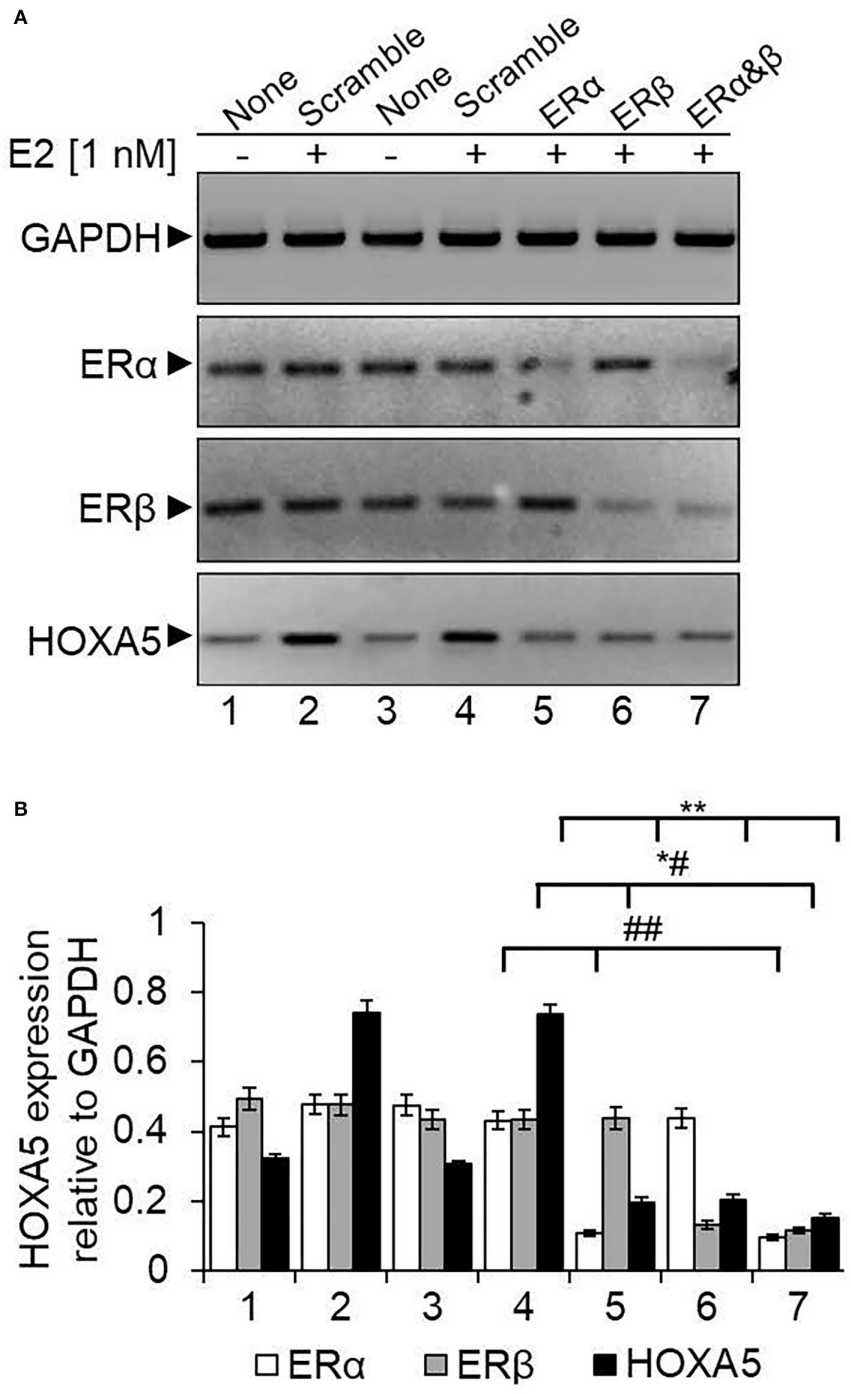
Knockdown of ERs and its impact on E2-induced HOXA5 expression. MCF7 cells were transfected with ERα or ERβ-specific ASOs or a scramble antisense for 48 h, followed by treatment with E2 (1 nM, 6 h). RNA was analyzed by RT-qPCR by using primers specific to HOXA5, ERs, and GAPDH (control) separately **(A)**. Lane 1: control cells (no E2); lane 2: cells treated with E2; lanes 3 and 4: cells treated with scramble antisense in the absence and presence E2; lanes 5 and 6: cells treated with ERα and ERβ antisense, respectively, followed by exposure to E2; lane 7: cells transfected with 1:1 mixture of ERα and ERβ antisenses in the presence of E2. The qPCR analysis data is in **(B)**. qPCR reactions were carried out in three parallel replicates, and each experiment was repeated at least thrice (*n* = 3). Bars indicate averages ± standard errors. **indicates *p* < 0.0001 compared to E2-treated scramble control (for HOXA5 target); *^#^indicates *p* < 0.0001 compared to E2-treated scramble control (for ERβ target); ^*##*^indicates *p* < 0.0001 compared to E2-treated scramble control (for ERα target).

ERs are crucial players in estrogen signaling (Nilsson and Gustafsson, 2000, 2002). In general, ERs are activated upon binding to E2. The activated ERs dimerize and bind to their target gene promoters. Along with ERs, various ER co-regulators and chromatin-modifying enzymes are also recruited and modify the chromatin and induce ER target gene expression (Nilsson et al., 2001; Nilsson and Gustafsson, 2002; Barkhem et al., 2004; Dreijerink et al., 2006; Lee et al., 2006; Mo et al., 2006). To understand the importance of ERs in HOXA5 gene regulation, we analyzed the HOXA5 expression in ER knocked down (using ERα or ERβ-specific ASO) and E2-treated MCF7 cells. As seen in Figure 5, ERα or ERβ levels were knocked down upon treatment with respective ASO (lanes 5–7, Figure 5A, qPCR data in Figure 5B). Interestingly, the level of E2-induced expression of HOXA5 was suppressed significantly upon knockdown of ERα or ERβ (lanes 5–7, Figures 5A,B). Scramble-ASO does not have any major impact on the E2-dependent HOXA5 expression (lanes 2–4, Figures 5A,B). The level of E2-induced HOXA5 expression is further suppressed by the combined knockdown of ERα and ERβ (lane 7, Figures 5A,B).

**Figure 6:**
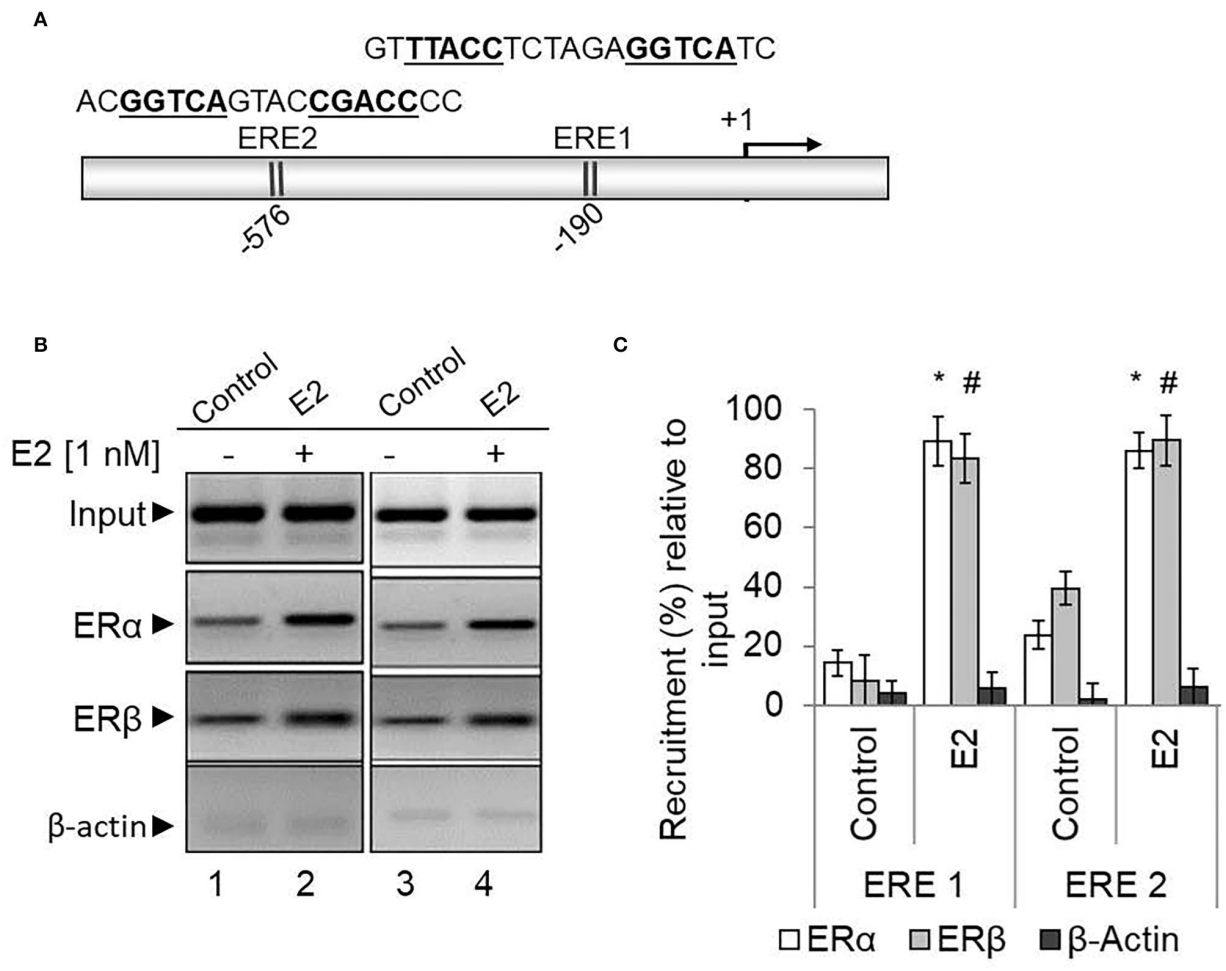
ERα and ERβ enrichment on the HOXA5 promoter ERE in the presence of E2. The HOXA5 promoter contains two putative ERE regions (ERE1 and ERE2) near the transcription start site **(A)**. E2-treated (nM E2 for 6 h) and control (untreated) MCF7 cells were analyzed by ChIP assay using antibodies against ERα, ERβ, and β-actin (control). The ChIP DNA was analyzed by regular PCR (agarose gel analysis, **B**) and qPCR **(C)** using HOXA5 promoter primers (ERE1 and ER2 regions). Bars indicate averages ± standard errors (*n* = 3). *denotes *p* < 0.0001 compared to ERα ChIP control; ^#^denotes *p* < 0.0001 compared to ERβ ChIP control for both the EREs.

As ERα and ERβ are associated with HOXA5 expression, we examined their binding to the HOXA5 promoter (Ansari et al., 2011b). We performed the ChIP assay using antibodies against ERα, ERβ, and β-actin (control), and the immunoprecipitated DNA were analyzed by PCR using primers spanning the estrogen-response element (ERE) regions of the HOXA5 promoter. Notably, promoter sequence analysis shows the presence of two potential EREs at the HOXA5 promoter upstream of the transcription start site (Figure 6A). ChIP analysis shows that levels of ERα and ERβ (but not β-actin) were enriched in the ERE1 and ERE2 regions of the HOXA5 promoter in the presence of E2 (Figure 6B, qPCR data in Figure 6C). Taken together, the antisense-mediated knockdown and ChIP analysis demonstrate the involvement of both ERα and ERβ HOXA5 gene expression in the presence of E2.

**Figure 7:**
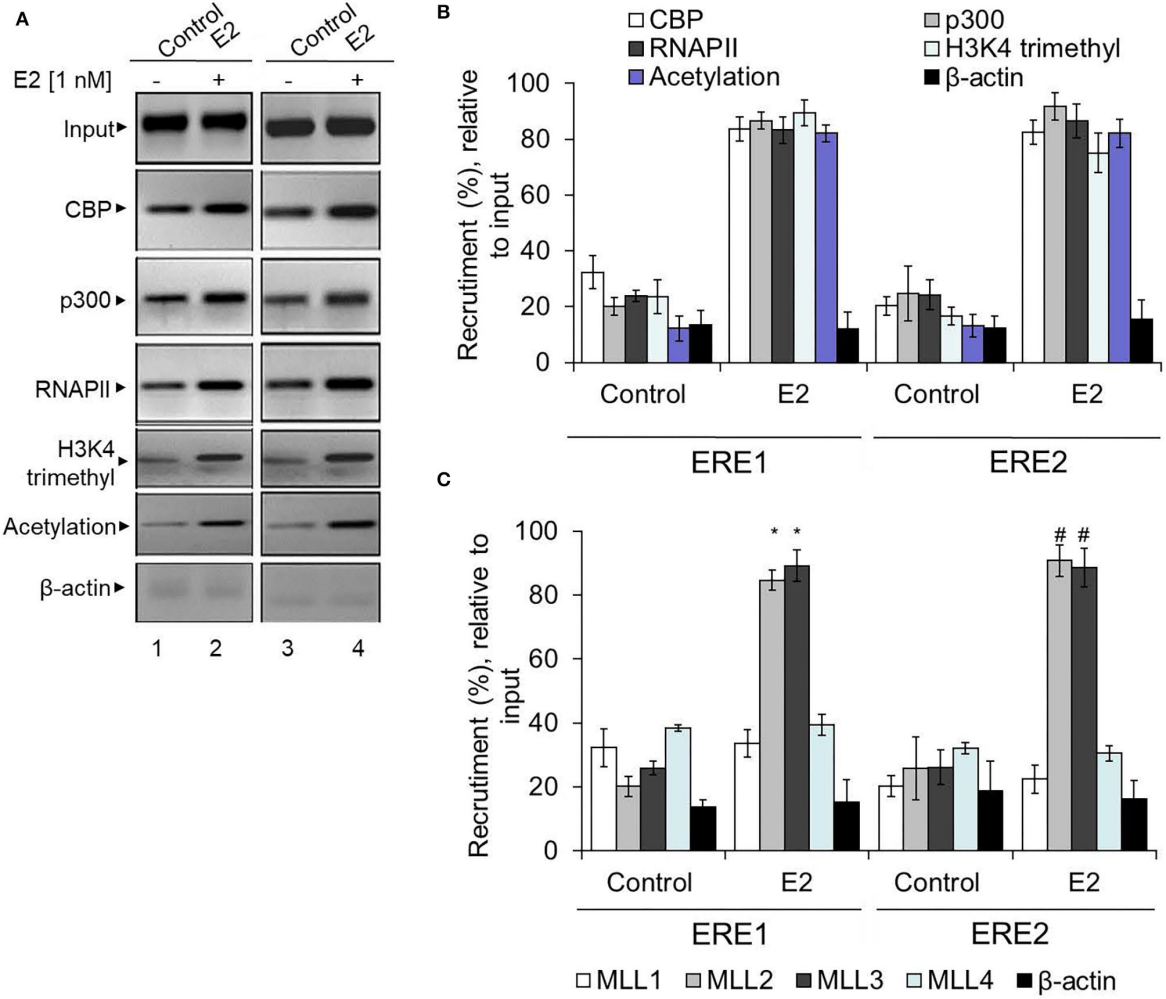
Enrichment of ER co-regulators at the HOXA5 promoter EREs. E2-treated (1 nM, 6 h) MCF7 cells were analyzed by ChIP assay using different antibodies. The ChIP DNA was analyzed by PCR using HOXA5 promoter primers (ERE1 and ERE2 regions). An agarose gel analysis showing the recruitment of selected factors on the ERE1 and ERE2 regions of the HOXA5 promoter is shown in **(A)**. qPCR analysis of the ChIP DNA samples showing the enrichment (relative to input) of CBP, p300, MLL1, MLL2, MLL3, MLL4, H3K4-trimethyl, histone acetylation, RNAP II, and β-actin into the ERE1 and ERE2 regions of the HOXA5 promoter region in the presence of E2 is shown in **(B,C)**. For **(B,C)**, bars indicate averages ± standard errors (*n* = 3). *denotes *p* < 0.0001 compared to respective ChIP control for ERE1; ^#^denotes *p* < 0.0001 compared to respective ChIP control for ERE2.

Along with ERs, ER co-activators are crucial players in ER target gene expression. Therefore, in addition to ERs, we analyzed the enrichment level of several well-known ER co-regulators: CBP/p300 (histone acetylase) and mixed lineage leukemias (MLL, histone methyltransferases) (Ansari et al., 2011b). Additionally, we also analyzed the level of histone acetylation and H3K4 trimethylation at the HOXA5 promoter using ChIP in the absence and presence of E2. Notably, promoter histone acetylation and H3K4 trimethylation are post-translational histone modifications associated with gene activation. Interestingly, our ChIP analysis shows that E2 treatment resulted in enrichment of CBP, p300, histone methylases MLL2 and MLL3 (Figures 7A–C). Histone acetylation, H3K4 trimethylation, and RNA polymerase II (RNAPII) levels were also elevated at the HOXA5 promoter with E2 treatments (Figures 7A,B). The E2-dependent enrichment in CBP/p300 acetyltransferase and MLL-histone methylases MLL2 and MLL3 and the histone acetylation and methylation levels, suggest that, along with ERs, these ER co-regulators are associated with E2-dependent HOXA5 gene expression.

## Discussion

HOX gene expression is well-known to guide cellular differentiation, organogenesis, and development (Krumlauf, 1994; Zakany and Duboule, 2007). Increasing studies demonstrate that hormones, such as estrogen, retinoic acid, etc., influence the expression of developmental genes, including HOX genes. Posterior HOX genes appear to be regulated by estrogens and progesterones, and anterior HOX genes are regulated by retinoids. HOXA5, which acts a transcription factor, plays a critical role in embryonic development. Beyond this, an emerging group of studies shows the dysregulation of HOXA5 in various solid/adult/pediatric/cancers or hematological malignancies and linked with higher pathological grade and poorer disease outcome (Chen et al., 2004; Wang et al., 2019). However, the detailed mechanism by which HOX genes influence differentiation and development and how it is regulated or misregulated in diseases/various carcinomas is still emerging and less understood. Here, we explore any association and function of HOXA5 in breast cancer and investigate its potential mechanism of transcription.

Our studies demonstrate that HOXA5 expression is also augmented in breast cancer tissues in comparison to the corresponding adjacent normal breast tissues. Additionally, c-bioportal-based meta-analysis of preexisting gene expression databases in breast cancer patients also show that HOXA5 expression is elevated in breast cancer patients. HOXA5 expression is also found to be elevated in ER-positive breast cancer cells. Additionally, our studies also demonstrate that HOXA5 expression is important for cell-cycle progression as well as cell viability of breast cancer cells. HOXA5 downregulation results in cell-cycle arrest and apoptosis in breast cancer cells. These observations suggest that HOXA5 expression is elevated in breast cancer at least in some subset of breast cancer and may be critical for breast cancer cell proliferation. As HOXA5 is elevated in ER-positive breast cancer cells and breast cancer tissues, we investigated its gene regulation potential via E2. These studies demonstrate that E2 indeed regulates the transcription of HOXA5 *in vitro*, in ER-positive breast cancer cells (MCF7), and this expression is suppressed upon treatment with an antiestrogen, tamoxifen, suggesting the potential regulation of HOXA5 via E2 and ERs. Additionally, we also observed that HOXA5 expression is elevated in the mammary tissues of OVX Sprague–Dawley rats, further supporting our observation that HOXA5 expression is regulated by estrogen *in vivo*.

Mechanistic studies demonstrate that E2-dependent HOXA5 expression is coordinated via involvement of ERs and ER co-activators. Knockdown of either ERα or ERβ suppressed E2-dependent HOXA5 expression in MCF7 cells. ChIP analysis demonstrates that ERs and ER co-activators, CBP/p300 (acetyl-transferases), and MLL2 and MLL3 (histone H3K4-methylases) are enriched at the HOXA5 promoter (ERE regions) in the presence of E2. Along with histone acetyl-transferases and histone methylases, the level of histone H3K4 trimethylation and histone acetylation level was elevated at the HOXA5 promoter. Notably, histone acetylation and H3K4 trimethylation are linked to gene activation. The enrichment of histone acetyl-transferases (CBP/p300) and histone H3K4 trimethylases (MLL2 and MLL3) and the histone acetylation and H3K4 trimethylations at the HOXA5 promoter in the presence of E2 suggest their important roles in E2-mediated regulation of HOXA5 expression in breast cancer.

**Figure 8:**
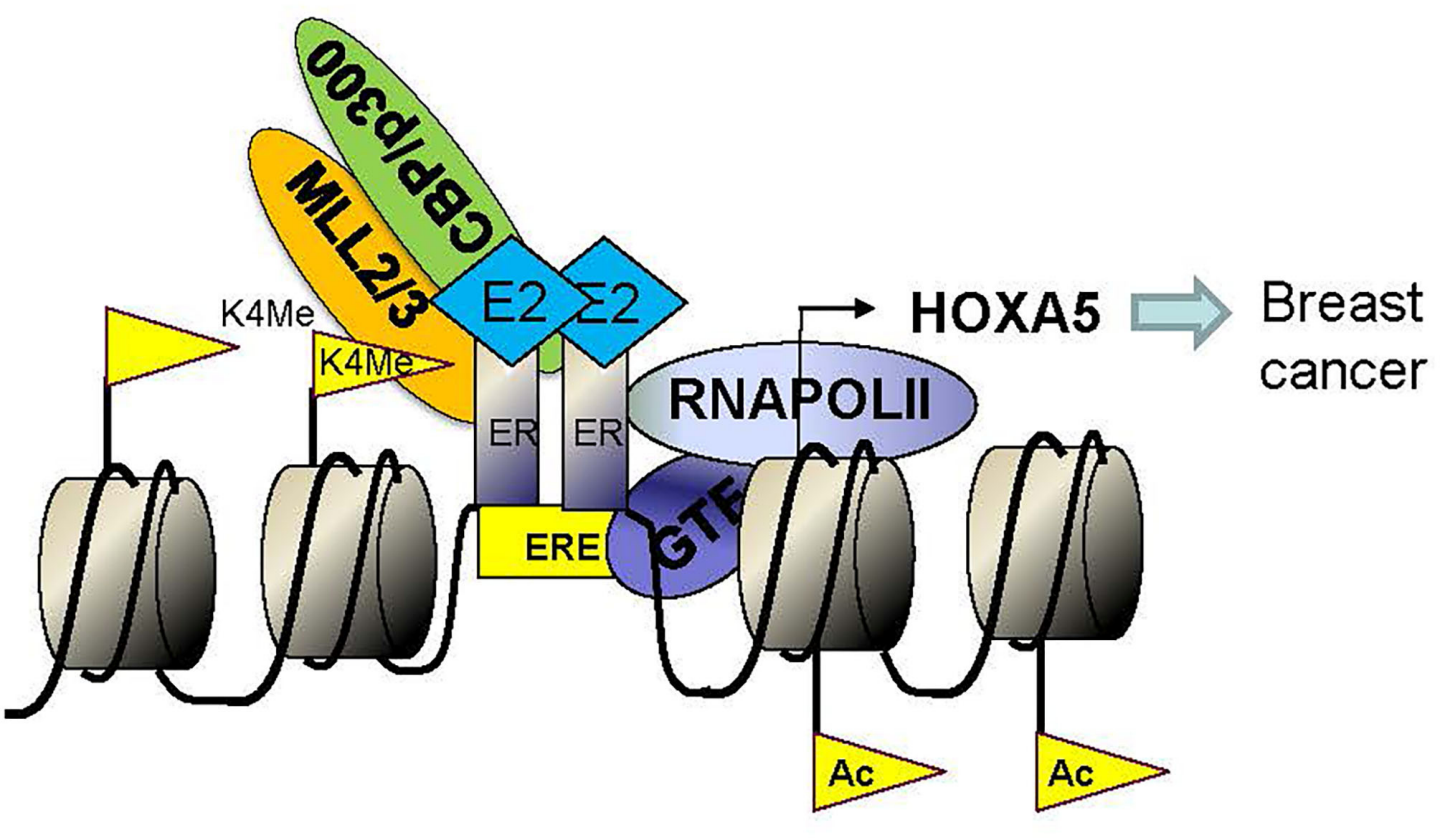
Model showing the mechanism of E2-mediated activation of HOXA5. Binding of E2 induces conformational change in ERs and induces dimerization and activation of ERs. Activated ER dimers enter the nucleus, bind to the HOXA5 promoter (EREs). ER co-regulators (CBP, p300, MLL2, MLL3, and others) also bind to the HOXA5 promoter, modify chromatins (H3K4-trimethylation via MLL2 and MLL3 and histone acetylation via CBP/p300), aid in recruitment of RNA polymerase II (RNAP II) and general transcription factors (GTFs) to the promoter and ultimately lead to transcription activation of HOXA5.

Overall, our studies demonstrate that HOXA5 gene expression is regulated by E2, and its expression is upregulated in breast cancers. The E2-mediated regulation of HOXA5 is coordinated via involvement of estrogen receptors, CBP/p300 histone acetyl-transferases, and the MLL family of histone methyl-transferases. CBP/p300 and MLL-histone methylases act as ER co-regulators in regulation of HOXA5 expression in breast cancer. A model showing the mechanism of E2-mediated HOXA5 gene activation is shown in Figure 8. Notably, multiples lines of evidence support our observations that increased H3K4-trimethylation and histone acetylation contribute to E2-induced HOXA5 expression. For example, Yan et al. demonstrate that inhibition of histone deacetylase HDAC8 (by HDAC inhibitor) increases histone acetylation and HOXA5 expression, which, in turn, induces tumor-suppressor p53 induction (Yan et al., 2013). Another study shows that oxidative stress–mediated repression of HDAC8 induces histone H3 acetylation and HOXA5 expression that controls plasticity of lung cancer stem-like cells (Saijo et al., 2016). Similar to histone acetylation, histone H3K4 trimethylation is well-known to activate HOX gene expression, including HOXA5 expression (Okada et al., 2006; Mishra et al., 2009; Ansari et al., 2012b; Burillo-Sanz et al., 2018). Enhancer elements present around the HOXA cluster regulates HOXA cluster gene spatiotemporal expression, and deletion impairs HOX5 gene activation in embryonic stem cells (Cao et al., 2017). Thus, our results showing the roles of H3K4 trimethylation and histone acetylation are in agreement with previous studies. Although histone acetylation and H3K4-trimethylation result in gene activation, H3K27 trimethylation by polycomb repressive complex (PRC) results in HOXA5 gene silencing and contributes to cell differentiation and development (Xi et al., 2007). Transcription factor CTCF facilitates the stabilization of PRC2 and H3K27 trimethylation at the HOXA locus and represses HOXA gene (including HOXA5) expression (Xu et al., 2014). Mutation in CTCF results in increased HOXA cluster gene expression. Similarly, ASXL1 mutation facilitates myeloid transformation through inhibition of PRC2-mediated HOXA5 repression (Abdel-Wahab et al., 2012). An independent study reports that HOXA5 expression is repressed in a subset of human breast cancer tissues, and this loss occurs via promoter hypermethylation (Teo et al., 2016). The study shows that HOXA5 loss in tumor cells causes reduced downstream target expression involved in maintaining epithelial integrity, which leads to a subsequent increase in invasion and migration. This suggests the potential presence of multiple modes of action of HOXA5 in different breast cancer subsets. Nevertheless, our studies demonstrate that HOXA5 expression is upregulated, at least in a subset of breast cancer, and is regulated by estrogen *in vitro* and *in vivo*, and therefore, may play critical roles in in breast cancer.

## Data Availability Statement

The raw data supporting the conclusions of this article will be made available by the authors, without undue reservation.

## Ethics Statement

The animal study was reviewed and approved by IACUC, University of Texas at Arlington.

## Author Contributions

IH did most of the experiments, performed the data analysis, and contributed to the manuscript writing. PD, AC, MO, KA, and AB assisted the different biochemical experiments. SB and LP coordinated the animal experiments. SU, PA, RB, and HD helped with the cancer-related studies. SM designed the experiments, supervised the study, and written the manuscript. All authors contributed to the article and approved the submitted version.

## Conflict of Interest

The authors declare that the research was conducted in the absence of any commercial or financial relationships that could be construed as a potential conflict of interest.
